# The prevalence of discrimination across racial groups in contemporary America: Results from a nationally representative sample of adults

**DOI:** 10.1371/journal.pone.0183356

**Published:** 2017-08-24

**Authors:** Brian B. Boutwell, Joseph L. Nedelec, Bo Winegard, Todd Shackelford, Kevin M. Beaver, Michael Vaughn, J. C. Barnes, John P. Wright

**Affiliations:** 1 School of Social Work, College for Public Health & Social Justice, Saint Louis University, 3550 Lindell Boulevard, St. Louis, Missouri, United States of America; 2 Department of Epidemiology, College for Public Health & Social Justice, Saint Louis University, St. Louis, Missouri, United States of America; 3 School of Criminology and Criminal Justice, University of Cincinnati, Cincinnati, OH, United States of America; 4 Department of Psychology, Florida State University, Tallahassee, FL, United States of America; 5 Department of Psychology, Oakland University, Rochester, MI, United States of America; 6 College of Criminology and Criminal Justice, Florida State University, Tallahassee, FL, United States of America; Macquarie University, AUSTRALIA

## Abstract

A large body of social science research is devoted to understanding the causes and correlates of discrimination. Comparatively less effort has been aimed at providing a general prevalence estimate of discrimination using a nationally representative sample. The current study is intended to offer such an estimate using a large sample of American respondents (N = 14,793) while also exploring perceptions regarding why respondents felt they were discriminated against. The results provide a broad estimate of self-reported discrimination experiences—an event that was only reported by about one-quarter of all sample members—across racial and ethnic categories.

## Introduction

Personal experiences of discrimination and bias have been the focus of much social science research. [1–3] Sociologists have explored the adverse consequences of discrimination [3–5]; psychologists have examined the mental processes that underpin conscious and unconscious biases [6]; neuroscientists have examined the neurobiological underpinnings of discrimination [7–9]; and evolutionary theorists have explored the various ways that in-group/out-group biases emerged across the history of our species. [10] In many respects, researchers already possess a wealth of knowledge concerning the origins and consequences of discrimination and bias. [11]

What also should not be lost in discussion of discrimination is the growing push to implement social policy aimed at reducing the occurrence of discriminatory practices. Mandatory diversity trainings in professional settings, for example, are intended to reduce bias in the workplace by increasing the awareness of employees regarding the challenges facing minority group members. [12] Indeed, the implementation of certain policies is rooted in the assumption that discrimination and biases are, at least to some appreciable amount, present in modern society.

Even so, estimates of the prevalence of perceived discrimination remains rare (see [13–14]). At least one prior study by Kessler and colleagues [15], however, using measures of perceived discrimination in a large American sample, reported that approximately 33% of respondents reported some form of discrimination (see also, Gibbons et al. [4]). The current study seeks to build on this research by estimating the prevalence of discrimination experiences among a large, nationally representative sample of adults from the United States. Additionally, the analysis address the perceived reasons for reported discrimination experiences.

## Methods

### Data

Data are derived from the National Longitudinal Study of Adolescent to Adult Health (Add Health). [16] Details about the sampling procedure are described elsewhere. [16] In brief, the Add Health is a nationally representative study of American youth who were followed into adulthood and who completed interviews at four time points. The first wave of data collection, Wave 1, occurred during the 1994–1995 school year. Wave 2 was conducted about one-and-a-half years later. After about five years, when the respondents were entering young adulthood, the third wave of data collection occurred. Last, during 2007 and 2008 the fourth wave of data collection was completed. At Wave 4, the respondents were approximately 29 years of age, on average (age range was 25 to 34 years). The Add Health study boasts strong respondent retention, with about 80% of Wave 1 respondents re-interviewed at Wave 4. [16] The current study employs data from Waves 1 and 4, which resulted in an analytical sample of N = 14,793. In accordance with Add Health guidelines [17], sampling weights for cross-sectional analyses were employed, as we will draw our analytical variables from the Wave 4 interview and only demographic details will be drawn from Wave 1. Note that because the outcome of interest (i.e., perceived discrimination) was derived from Wave 4, all estimates were weighted according to information available in the Add Health sampling weight variable *GSWGT4_2*. Moreover, the clustering of respondents within schools at Wave 1 (*PSUSCID*) and the stratification by region (*REGION*) that was used to define the sampling frame were taken into account when calculating standard errors (see [17]).

### Measures

#### Perceived discrimination

The current study employed two measures of perceived discrimination derived from a single question asked during the Wave 4 interview (see also [18–19]; as well as [15] and the work of Pang [20] who analyzed discrimination measures from Wave I of the Add Health in order to predict various outcomes among the participants). The first measure reflects responses to the following question: “In your day to day life, how often do you feel you have been treated with less respect or courtesy than other people?” The responses to this question were coded such that 0 = *never*, 1 = *rarely*, 2 = *sometimes*, and 3 = *often*. Note that in comparison to Everett et al. [19] who restrict their analysis to the dichotomous measure of perceived discrimination in the Add Health data, our analyses employed both the dichotomous version and the categorical version of the measure. Additionally, while Everett et al.’s [19] analyses were focused on the impact of discrimination experiences on mental health our analyses are centered primarily on a general assessment of the overall incidence of such experiences.

The second measure is a dichotomized version of the above measure, coded such that 0 = *never/rarely* and 1 = *sometimes/often*. The descriptive statistics for the variables included in the current study are presented in Table 1.

**Table 1:** Descriptive statistics of the study variables and racial categories for all respondents in the analytical sample.

	N	Minimum	Maximum	Mean	SD
Study Variables					
Discrimination experiences	14,793	0	3	.99	.83
Ever experience discrimination	14,793	0	1	.25	.43
Reason for discrimination ^a^	3,613	1	9	9	—
Age	14,783	25	34	28.95	1.82
Sex (0 = female, 1 = male)	14,793	0	1	.51	.50
Racial Categories	Frequency	Proportion			
White	9,707	66.31			
Black	2,221	15.17			
Hispanic	1,578	10.78			
American Indian	126	.86			
Asian	445	3.04			
Mixed Race	561	3.83			
N (non-missing on racial category)	14,638	100			

Discrimination variables, age, and sex are from Wave 4, the racial categories are from Wave 1; the cross-sectional sampling weight variable from Wave 4 was employed to estimate values.

^a^Modal category displayed as mean.

#### Reason for discrimination

All respondents who indicated they were discriminated against—specifically, those who responded with *sometimes* or *often* to the perceived discrimination measure described above—were asked the following question: “What do you think was the main reason for these experiences?” Respondents were allowed to choose *one* response from 11 categories. For the present analysis, these responses were recoded into nine mutually exclusive categories capturing the following options: 1) race/ancestry/skin color; 2) gender; 3) age; 4) religion; 5) height or weight; 6) sexual orientation; 7) education or income; 8) physical disability; and 9) other. The following categories from the original questionnaire were collapsed into one category for the analysis: race; ancestry or national origin; and shade of skin color. Additionally, because this question was only asked of respondents who reported prior discrimination experiences, the built-in skip pattern resulted in a large number of cases scored as missing (*legitimate skip*).

#### Respondent race

Because a race variable is not available from the Wave 4 interviews, we use the racial category reported by the respondent during the Wave 1 interview. Wave 1 race—rather than, say, Wave 3 race—was used to preserve case counts. The logic is that (nearly) all Wave 4 respondents appeared in the Wave 1 sample, but not all would have been interviewed at Wave 3 due to differential patterns of temporary attrition. Respondents were asked to indicate their race from among the following categories: White; Black or African American; Hispanic; American Indian or Native American; and Asian or Pacific Islander. Respondents were provided the opportunity to select more than one race, and those who did were asked a follow-up question regarding which category best described their racial background. In the current study, those respondents who indicated more than one race were coded as “mixed race”.

#### Demographic variables

To provide information on the analytical sample as a whole, two additional demographic variables are included. First, age is a continuous measure created by subtracting the year of the respondents’ birth (obtained from Wave 1) from the year of the interview at Wave 4. Second, sex was dichotomously coded based on the self-reported sex of the respondent at Wave 4 (0 = *female* and 1 = *male*).

### Analytical plan

Our exploratory study included three basic steps. First, summary statistics of the study variables and racial categories were produced. Second, we examined the relative proportions of the two *discrimination experience* measures across each racial category. Finally, we assessed the distribution of reported *reasons for discrimination* across the racial categories. In order to examine potential bivariate associations, the adjusted *F* statistic (design-based *F*) was employed as it corrects for a complex sample design such as that used in the Add Health. [21] More specifically, when analyzing weighted sample data employing the svy suite of commands in Stata the conventional Pearson *χ*^2^ statistic test of independence is converted into “an *F* statistic with noninteger degrees of freedom by using a second-order Rao and Scott (1981[22], 1984[23]) correction”. [24] The *p*-value associated with the design-based *F* is thus more accurate (than the *p*-value associated with the *χ*^2^ statistic) given the adjustments and calculations take into account the weighted nature of the data. This final step also included an examination of the relative distribution of racial categories across the various reported *reasons for discrimination*. As noted earlier, all analyses were weighted according to the survey weight provided by the Add Health research staff and standard errors were corrected for the clustering and stratification that defined the sampling strategy. Thus, all estimates reported here can be considered nationally representative of the United States.

## Results

Table 2 reveals the frequencies at which respondents in the Add Health reported experiencing discrimination. Beginning broadly, it appears that most of the respondents (about 75%) reported either having *never*, or *only rarely*, been discriminated against in their day-to-day lives. Individual responses can be observed across racial categories, with this same pattern of discrimination experiences reported within the different racial categories. For all racial and ethnic groups represented in the data, the majority reported experiencing either none or infrequent discrimination. However, note that there appears to be a statistically significant difference between the racial categories and reported discrimination experiences (Design-based *F*_(11.69, 1496.80)_ = 4.16, *p* < .0001) and ever experiencing discrimination (Design-based *F*_(4.68, 598.86)_ = 8.73, *p* < .0001).

**Table 2:** Relative proportions of discrimination experiences by racial category.

		Discrimination Experiences^a^						Ever Experience Discrimination^b^	
		Never	Rarely	Sometimes	Often	Total		No	Yes
White (N = 9,707)	Row %	30.34	46.13	19.41	4.12	100		76.47	23.53
	Column %	66.13	68.93	61.69	62.94	66.31		67.80	61.90
Black (N = 2,221)	Row %	29.83	38.29	26.46	5.41	100		68.12	31.88
	Column %	14.88	13.09	19.25	18.92	15.17		13.82	19.19
Hispanic (N = 1,578)	Row %	32.52	40.33	22.14	5.01	100		72.85	27.15
	Column %	11.53	9.80	11.44	12.43	10.78		10.50	11.61
American Indian (N = 126)	Row %	25.10	47.88	22.13	4.89	100		72.98	27.02
	Column %	0.71	0.93	0.92	0.97	0.86		0.84	0.93
Asian (N = 445)	Row %	34.24	47.04	16.90	1.81	100		81.28	18.72
	Column %	3.43	3.23	2.47	1.27	3.04		3.31	2.26
Mixed Race (N = 561)	Row %	26.37	46.64	23.07	3.93	100		73.01	26.99
	Column %	3.32	4.03	4.24	3.47	3.83		3.74	4.11
Total (N = 14,638)	Row %	30.42	44.38	20.86	4.34	100		74.80	25.20
	Column %	100	100	100	100	100		100	100

**T**he cross-sectional sampling weight variable from Wave 4 was employed to estimate values.

^a^Design-based *F*_(11.69, 1496.80)_ = 4.16, *p* < .0001

^b^Design-based *F*_(4.68, 598.86)_ = 8.73, *p* < .0001

Table 3 provides an in-depth assessment of the *perceived reasons for discrimination* reported by those who *sometimes* or *often* experienced discrimination. The vast majority of these respondents reported the discrimination was due to reasons other than those covered in the nine mutually exclusive categories. Thus, the most common explanation was *not* due to race, gender, sexual orientation, or age. Instead, the vague category of *other* seems to best describe the perceived source of the average American’s discrimination experiences

**Table 3:** Reason given by respondents for discrimination for the total (restricted) sample and by racial category.

	Total^a^	White		Black		Hispanic		American Indian		Asian		Mixed Race	
Reported Reason	Col.%	Row%	Col.%	Row%	Col.%	Row%	Col.%	Row%	Col.%	Row%	Col.%	Row%	Col.%
Race/Ancestry/Skin color	10.38	21.56	3.62	46.99	25.41	22.78	20.40	0.41	4.55	3.78	17.09	4.48	11.22
Gender	4.71	69.86	5.32	16.88	4.14	5.64	2.29	0.21	1.07	3.17	6.51	4.23	4.81
Age	7.50	68.34	8.29	11.35	4.43	13.43	8.68	0.05	0.43	2.79	9.11	4.04	7.31
Religion	0.88	51.28	0.73	43.75	2.01	3.76	0.29	0.00	0.00	1.11	0.43	0.01	0.02
Height or weight	5.47	75.91	6.71	12.71	3.62	4.58	2.16	0.00	0.00	0.93	2.23	5.86	7.74
Sexual orientation	0.39	58.79	0.37	26.19	0.53	11.43	0.38	0.00	0.00	3.22	0.54	0.37	0.03
Education or income	8.97	58.05	8.42	19.36	9.05	14.57	11.27	1.07	10.17	1.34	5.22	5.61	12.15
A physical disability	3.04	63.52	3.12	15.20	2.41	10.80	2.83	0.00	0.00	0.00	0.00	10.47	7.68
Other	58.66	66.83	63.41	15.84	48.40	10.22	51.70	1.34	83.79	2.30	58.87	3.46	49.04
N (Row%)	3,613 (100)	2,234 (61.83)		694 (19.20)		419 (11.59)		34 (.94)		83 (2.29)		150 (4.14)	

Only individuals who indicated that they had experienced some form of discrimination provided a reported reason; a large number of cases are missing on the Reported Reason variable (due to *legitimate skips*) which accounts for the difference in *n* for the racial categories from other tables; the cross-sectional sampling weight variable from Wave 4 was employed to estimate values.; “Col.”: Column.

^a^Design-based *F*_(18.86, 2414.24)_ = 6.26, *p* < .0001

**Fig 1:**
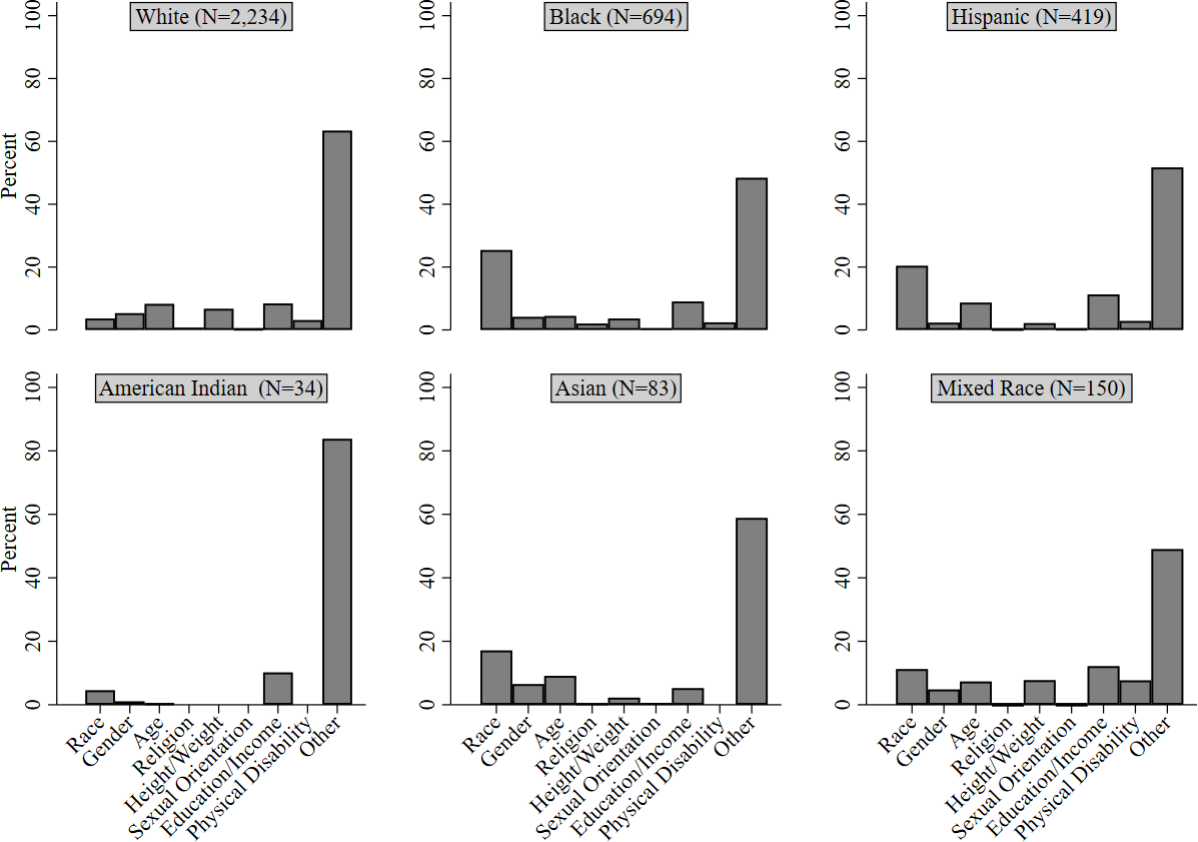
Sample weighted estimated proportion of reasons for discrimination for each racial category (see Table 3).

As for the remainder of the responses in the total sample, discrimination owing to race/ancestry/skin color was the most commonly reported cause (around 10%), and was followed closely by economic/educational factors (around 9%), and age (around 7%). Fig 1 presents the findings of Table 3 in graphical format.

## Discussion

Using a representative sample of American respondents who reflect a variety of racial and ethnic groups, the current study examined perceived experiences of discrimination. Our results indicate that the majority of the sample reported either no experience with discrimination or that it had happened only rarely. Moreover, of those reporting having experienced discrimination, the majority suggested that unique and perhaps situationally specific factors *other than* race, gender, sexual orientation, and age were the cause(s) of discrimination (for additional insight, see Everett et al. [19]). Our results thus provide at least somewhat of a counterweight to possibly exaggerated claims that discrimination is a prevalent feature of contemporary life in the United States. Results from the Add Health data seem generally inconsistent with such claims.

Prior to concluding, it is important to highlight limitations of the current study. First, not all racial and ethnic groups represented in the United States were included in the sample. Second, the measure of discrimination included in the Add Health data was quite broad and could capture acts that were not necessarily discriminatory but rather unfair in nature. In turn, such a conceptualization on the part of respondents could be one reason for the preponderance of the *other* category as a perceived reason for the discrimination (or unfair) experience. If discrimination were operationalized in a different manner, one might expect a different pattern of findings. The “experiences of discrimination” (EOD) measure analyzed by Krieger and colleagues [13], for example, is a multi-item construct that has been shown to be a reliable and valid assessment of self-reported racial discrimination. Importantly, single item indicators of discrimination seem to perform poorly when compared to multi-item measures. [13] As a result, we might have uncovered higher levels of self-reported discrimination if we had used a multi-item measure like the EOD. The Add Health did not include these items, yet the Add Health measures have been used to study the impact of discrimination experiences (e.g., Everett et al. [19]). For additional discussion regarding the use of self-reported perceived discrimination measures in US samples, see also Kessler and colleagues. [15]

In short, much caution is necessary when interpreting our findings. What should be avoided is the conclusion that our results suggest that the problem of discrimination in the US is, to any great extent, remedied and in need of no further scrutiny or improvement. Indeed, the observation of a bivariate association between the racial categories and discrimination experiences (see Tables 2 and 3) suggests that such experiences vary by race, which should remain a topic of focus for social and behavioral scientists, as well as for members of the community (although perhaps not in ways hitherto suspected).

An additional point to consider is that although the Add Health data contained a number of possible reasons why someone might feel discriminated against, there were still a number of additional reasons that were not covered in our analyses. For instance, someone who perceived discrimination because of their political affiliation, a form of discrimination that might be especially likely to be perceived during times of political polarization [25], would be captured in the “other” category. Our study, however, was unable to address these other possible sources of discrimination with any degree of clarity. Moreover, individuals might perceive that discrimination against their group exists and is a problem (i.e., against the race or ethnicity with which they self-identify), yet they do not feel that they, themselves, have been disrespected or discriminated against. Had the question been phrased differently, the response pattern may have been different. Moreover, while our analyses were distinguished by racial category, this is not the only way in which discrimination experiences can be differentiated. For example, if our analyses were conducted based on sex or sexual orientation, the results may differ. For the sake of brevity, our analyses were limited to racial differentiation and we recognize that future research employing an alternate approach may generate different results.

Finally, respondents were *not* instructed to bound their responses to a particular time frame (such as discrimination experienced in the last week, month, or year) and, therefore, the item should reflect *any* experience with discrimination. This could be problematic if participants failed to recall instances of discrimination, and thus underreported their experiences. Alternatively, an unbounded question prompt does not restrict discrimination experiences to an arbitrarily defined time period. While this might have some desirable properties (widening the net of possible experiences to be included as “discriminatory”), it might also serve to distort prevalence estimates in a variety of other ways (i.e., forgetting instances, or misremembering them, etc.). We await replications of our findings.
